# Aortoduodenal Fistula With IgG4-Related Periaortitis: A Case Report

**DOI:** 10.7759/cureus.73193

**Published:** 2024-11-07

**Authors:** Tatsunori Shizuku, Hiroyuki Yamaguchi

**Affiliations:** 1 Internal Medicine, US Naval Hospital Okinawa, Okinawa, JPN; 2 Rheumatology, Funabashi Municipal Medical Center, Funabashi, JPN

**Keywords:** abdominal aortic aneurysms, duodenal fistula, endovascular stenting, endovascular surgical repair, igg 4 disease

## Abstract

A 77-year-old woman with a history of endovascular aneurysm repair (EVAR) for an abdominal aortic aneurysm (AAA) presented with melena. She had been recently diagnosed with IgG4-related periaortitis and started on prednisone. Physical examination revealed pallor conjunctiva and melena on the rectal examination, with laboratory results indicating anemia (hemoglobin: 7.4 g/dl). Abdominal CT showed air within the aneurysmal sac, and endoscopy confirmed an aortoduodenal fistula. The patient underwent urgent stent removal, vessel replacement, and duodenal repair. She recovered well and was discharged on day 30 with ongoing prednisone therapy. The risk of fistula formation in IgG4-related periaortitis necessitates careful monitoring, especially in patients with pre-existing aortic pathology.

## Introduction

IgG4-related disease is an immune-mediated condition characterized by a fibroinflammatory process; it can involve any organ, most commonly the pancreas, salivary glands, and lacrimal glands [1]. Large vessel involvement, including periaortitis and aortitis, occurs in approximately 20% of cases of IgG4-related disease [2]. IgG4-related periaortitis specifically affects the aorta and its adjacent tissues, often presenting as significant thickening of the perivascular tissue on radiological exams. There is a potential risk of aortic rupture following the initiation of steroid therapy, especially if luminal dilatation is present before treatment [3]. We report a case that underscores the necessity of vigilant monitoring for the progression of the aneurysm in patients following the initiation of steroid treatment.

## Case presentation

A 77-year-old Asian female, who had undergone endovascular aneurysm repair (EVAR) for an abdominal aortic aneurysm (AAA) five years prior, presented with melena and was brought to the emergency department. She had presented to our hospital a month ago with an unexplained fever persisting for several weeks. At that time, the only significant findings after an extensive evaluation, including serologic testing, imaging studies, and an infectious workup were elevated serum IgG4 levels (145 mg/dL) and notable wall thickening of the aorta, involving the surrounding adjacent tissues. She had been diagnosed with an inflammatory abdominal aortic aneurysm (IAAA) due to IgG4-related periaortitis and started on 60 mg/day of prednisone two weeks ago.

**Figure 1:**
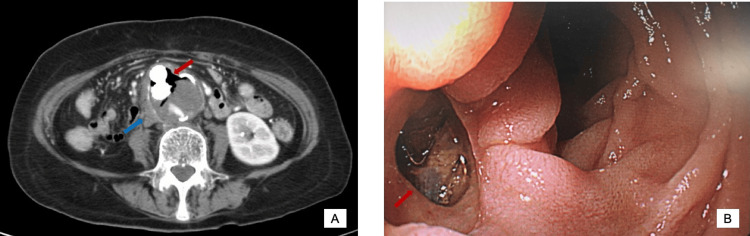
Contrast-enhanced CT of the abdomen (A) and upper gastrointestinal endoscopy (B) A: The image reveals air within the aneurysm (red arrow) and thickening of the aortic wall (blue arrow). B: The image of upper gastrointestinal endoscopy in the descending duodenum indicates an aortoduodenal fistula and the stent visible through the fistula (red arrow) CT: computed tomography

Upon presentation, her vital signs were as follows: temperature: 36.3 °C, heart rate: 99 beats per minute, and blood pressure: 99/50 mmHg. Physical examination revealed conjunctival pallor and melena on the rectal examination. Laboratory tests showed anemia with a hemoglobin level of 7.4 g/dl. A contrast-enhanced CT of the abdomen identified air within the aortic aneurysm (Figure 1A) and urgent upper gastrointestinal endoscopy revealed an aortoduodenal fistula with the stent visible through the fistula (Figure 1B). The patient immediately underwent removal of the stent graft, artificial vessel replacement, and duodenal closure with omental implantation. She recovered uneventfully and was discharged on the 30th day after admission while continuing treatment with prednisone. She remained stable on a low dose of prednisone (5 mg/day) at her one-year follow-up.

## Discussion

Aortoduodenal fistula is the most common type of enteric-aortic fistula, primarily due to the anatomical proximity of the duodenum to the aorta [3]. Risk factors for aortoduodenal fistula include prior aortic surgery, aortic aneurysms, infections, inflammatory conditions, radiation, malignancies, and connective tissue disorders [3]. Primary aortoduodenal fistula is exceedingly rare, typically arising postoperatively; however, it has been proposed that IgG4-related aortitis/periaortitis may contribute to fistula formation [3].

IgG4-related disease is an idiopathic condition characterized by infiltration of lymphocytes and plasma cells with associated fibrosis, resulting in organ enlargement, nodules, or thickened lesions in various tissues [4]. This disease can also involve fibrosis of the aorta and retroperitoneum. In the present case, the presence of elevated serum IgG4 levels (145 mg/dL) and imaging studies showing retroperitoneal fibrosis and wall thickening of the aorta supported a diagnosis of "possible" IgG4-related disease, leading to the initiation of steroid therapy.

Glucocorticoids are the first-line treatment for IgG4-related disease and have demonstrated efficacy [4]. However, there is a reported risk of exacerbation of aortic dilation and potential rupture following the initiation of steroid therapy, particularly if aortic luminal dilation exists before treatment [5]. Therefore, in patients with inflammatory aortic aneurysms due to IgG4-related disease, the decision to commence glucocorticoid therapy should be made cautiously, with careful monitoring for aneurysmal progression following treatment initiation.

## Conclusions

We presented a case of an aortoduodenal fistula in a patient with IgG4-related periaortitis. Vigilant monitoring for aneurysm progression and formation of fistula after the initiation of steroid treatment is important in patients suspected of having IgG4-related periaortitis.
